# Minimally invasive surgical repair for unroofed coronary sinus syndrome directed by three-dimensional transesophageal echocardiography

**DOI:** 10.1186/s40792-020-00978-8

**Published:** 2020-10-01

**Authors:** Kazuma Handa, Shinya Fukui, Mutsunori Kitahara, Yumi Kakizawa, Hiroyuki Nishi

**Affiliations:** Department of Cardiovascular Surgery, Osaka General Medical Center, 3-1-56 Bandai-Higashi, Sumiyoshi, Osaka 556-8558 Japan

**Keywords:** Unroofed coronary sinus syndrome, Minimally invasive cardiac surgery, Three-dimensional transesophageal echocardiography, Congenital heart disease

## Abstract

**Background:**

The recent remarkable development of cardiac imaging technology for unroofed coronary sinus syndrome has led to accurate preoperative diagnosis. We report a case of unroofed coronary sinus syndrome repaired via a minimally invasive approach, under the excellent command of three-dimensional transesophageal echocardiography.

**Case presentation:**

A 77-year-old woman with hypertension was admitted for aggravation of bilateral leg edema and diagnosed with type III unroofed coronary sinus syndrome with Qp/Qs ratio of 1.6:1. The unroofed portion was detected at the atrial side between P2 and P3 of posterior mitral leaflet by preoperative three-dimensional transesophageal echocardiography. Right minithoracotomy was performed at the fourth intercostal space and cardiopulmonary bypass routinely established. Right atriotomy and left atriotomy incisions were made under antegrade cardioplegic arrest. The unroofed portion was revealed at the same location by preoperative transesophageal echocardiography and was clearly recognized only by endoscopy, not by direct vision. It was repaired by direct running suture under endoscopic visualization. We observed no blood cardioplegia leakage or mitral insufficiency, which was also confirmed by postoperative transesophageal echocardiography. The patient’s postoperative course was uneventful and she was discharged home 14 days after surgery without any residual shunt.

**Conclusions:**

Successful repair of unroofed coronary sinus syndrome was safely and effectively achieved by a minimally invasive approach supported by preoperative three-dimensional transesophageal echocardiography.

## Background

Unroofed coronary sinus syndrome (URCS) is a complex subset of atrial septal defect (ASD), the traditional standard treatment of which was intracardiac repair by median sternotomy. Nowadays, minimal invasion presents an alternative approach to conventional cardiac surgery. However, using this approach to repair URCS must surmount the problem of limited intraoperative detection of the lesion by an endoscope. Moreover, preoperatively it is often difficult to detect the exact location of the unroofed portion, its anatomical relationship with the orifice of coronary sinus (CS), and the coexistence of other cardiac abnormalities. Recently, remarkable developments in cardiac imaging technology for URCS have enabled accurate preoperative diagnosis and detection of the precise location of the unroofed portion. We report a case of URCS repair via a minimally invasive approach using preoperative three-dimensional (3D) transesophageal echocardiography (TEE).

## Case presentation

**Fig. 1:**
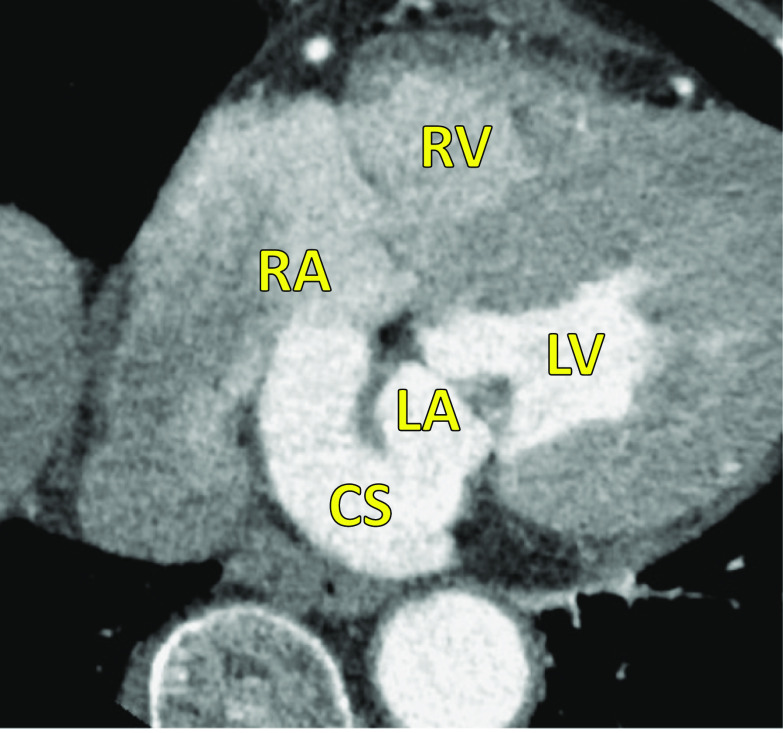
Preoperative cardiac computed tomography. Preoperative cardiac computed tomography showed extensive communication between the right and left atria caused by partial absence of the roof of the coronary sinus. *LA* left atrium, *LV* left ventricle, *RA* right atrium, *RV* right ventricle, *CS* coronary sinus

**Fig. 2:**
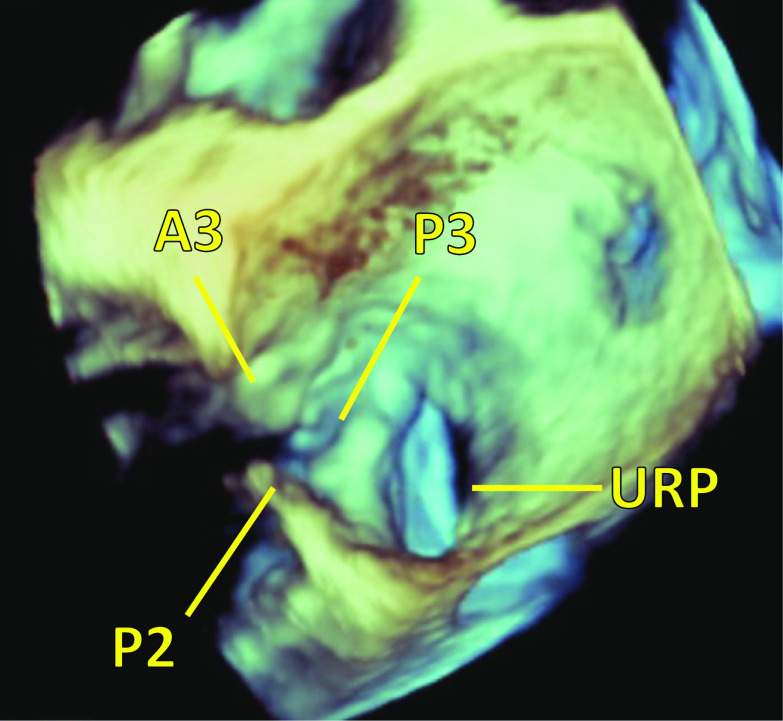
Preoperative three-dimensional transesophageal echocardiography. Preoperative three-dimensional transesophageal echocardiography showed there was an unroofed portion (20 × 10 mm) at the atrial side between segments P2 and P3 of the posterior mitral leaflet. *P2* P2 segment of posterior mitral leaflet, *P3* P3 segment of posterior mitral leaflet, *A3* A3 segment of anterior mitral leaflet, *URP* unroofed portion

A 77-year-old woman with hypertension was admitted for aggravation of bilateral leg edema and diagnosed with type III URCS by TEE, cardiac computed tomography (CT) (Fig. 1), and cardiac magnetic resonance imaging (MRI). Moderate tricuspid valve regurgitation was also detected by echocardiography. She had previously undergone endoscopic dissection for sigmoid cancer 9 years previously. Electrocardiography revealed sinus rhythm, and chest radiography showed a slight cardiomegaly. 3D TEE showed an unroofed portion (20 × 10 mm) at the atrial side between segments P2 and P3 of the posterior mitral leaflet (Fig. 2). Color Doppler imaging revealed shunt flow from the left atrium to the right atrium though the unroofed portion with moderate tricuspid regurgitation. Cardiac CT and MRI also showed findings of URCS without persistent left superior vena cava (PLSVC) or other cardiac abnormalities. Cardiac catheterization revealed the left-to-right shunt with Qp/Qs ratio of 1.6:1, and selective coronary angiography verified normal coronary arteries.

**Fig. 3:**
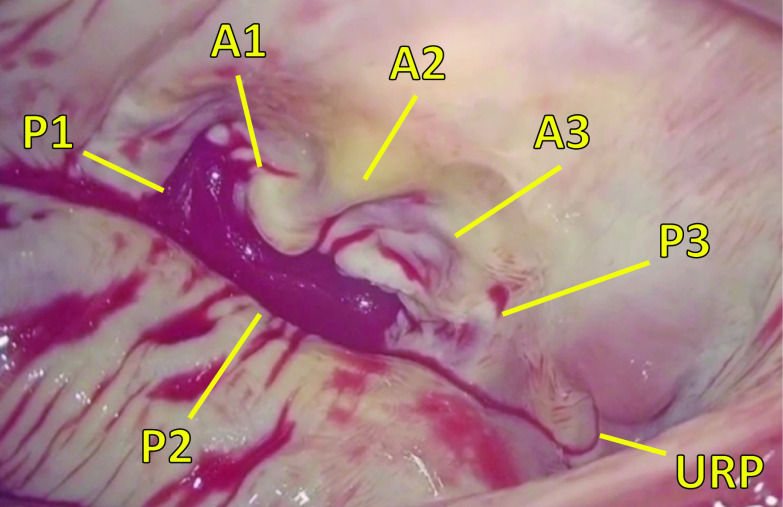
Intraoperative findings of the unroofed portion. Intraoperative findings showed the unroofed portion detected at the atrial side between segments P2 and P3 of the posterior mitral leaflet, similar to findings of preoperative transesophageal echocardiography. *P1* P1 segment of posterior mitral leaflet, *P2* P2 segment of posterior mitral leaflet, *P3* P3 segment of posterior mitral leaflet, *A1* A1 segment of anterior mitral leaflet, *A2* A2 segment of anterior mitral leaflet, *A3* A3 segment of anterior mitral leaflet, *URP* unroofed portion

**Fig. 4:**
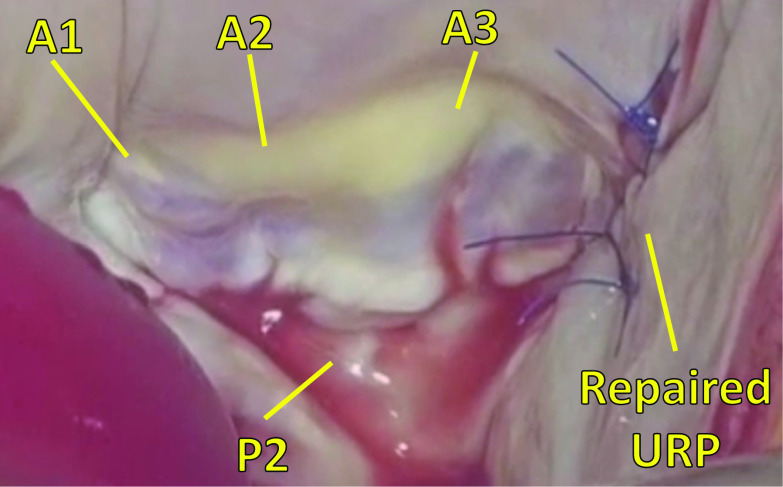
Intraoperative photo showing completion of the unroofed portion repair. Intraoperative photo showing completion of repair of the unroofed portion by use of a direct running suture from the top and bottom edges. *P2* P2 segment of posterior mitral leaflet, *A1* A1 segment of anterior mitral leaflet, *A2* A2 segment of anterior mitral leaflet, *A3* A3 segment of anterior mitral leaflet, *URP* unroofed portion

**Fig. 5:**
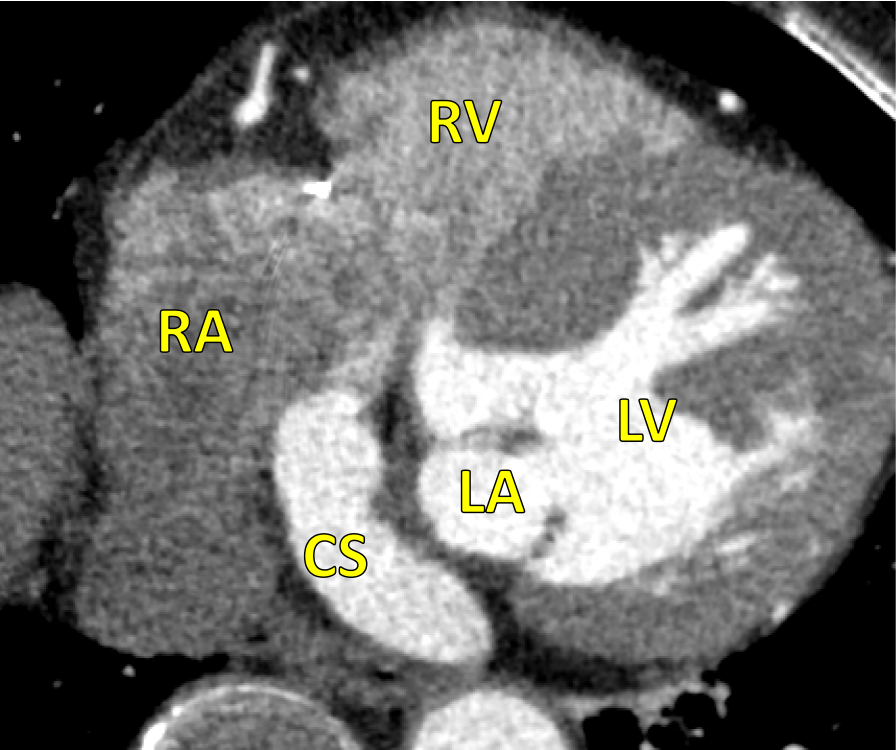
Postoperative cardiac computed tomography. Postoperative cardiac computed tomography image showing successful repair of the unroofed coronary sinus without any residual shunt. *LA* left atrium, *LV* left ventricle, *RA* right atrium, *RV* right ventricle, *CS* coronary sinus

An operation was performed using a right minithoracotomy at the fourth intercostal space, whereby a 6-cm-long skin incision in the anterior axillary line was made and a service port for the endoscope was opened through the same intercostal space at the mid-axillary line. We used a 3D full HD telescope (TIPCAM^®^1 S 3D, KARL STORZ SE & Co. KG, Tuttlingen, Germany) in the present case. Cardiopulmonary bypass was routinely established by right internal jugular vein and right femoral vessel cannulation, and right atriotomy and left atriotomy incisions were made under antegrade cardioplegic arrest. The unroofed portion, where the blood cardioplegia was leaking, was detected at the same location revealed by the preoperative 3D TEE (Fig. 3). The lesion was clearly recognized only by the endoscope. In the surgical field obtained by use of a left atrium retractor for right mini-thoracotomy, the medial side of the left atrium is usually unfolded. As a result, it was difficult to recognize the unroofed coronary sinus directly in the present case, because it was parallel to the field of view. The unroofed portion could be clearly confirmed only by changing the angle of the thoracoscope. We couldn’t identify the unroofed portion through the CS on the right atrium. It was repaired by direct running suture using 4-0 polypropylene sutures from the top and bottom edges under endoscopic visualization (Fig. 4). We confirmed that there was no blood cardioplegia leakage and mitral insufficiency, which was also verified by postoperative TEE. Operative findings showed that the tricuspid annulus was dilated and we finally performed a tricuspid annuloplasty with use of a 28-mm Carpentier-Edwards Physio tricuspid ring (Edwards Lifesciences, Irvine, CA, USA) with bicaval snaring. The patient’s postoperative course was uneventful, and she was discharged home 14 days after surgery without any residual shunt as confirmed by echocardiography and cardiac CT (Fig. 5).

## Discussion

URCS has been classified into four morphological types: type I, completely unroofed with PLSVC; type II, completely unroofed without PLSVC; type III (as in the current case), partially unroofed in the midsection; and type IV, partially unroofed in the terminal portion [1]. It is often difficult to detect preoperatively the precise unroofed portion, its anatomical relationship with the orifice of CS, and the coexistence of other cardiac abnormalities. For this reason, standard treatment has consisted of intracardiac repair by median sternotomy. Today, however, the minimally invasive approach has become a favored alternative to the conventional one in cardiac surgery, bringing better cosmetic outcome, postoperative recovery, and improved quality of life to patients. To our knowledge, in addition to our present case the literature has reported three cases of URCS repair via a minimally invasive surgical approach (Table 1) [2–4]. Among these cases, one patient and our patient were diagnosed as type III URCS and two as type IV, while one had multiple abnormality with secundum ASD [4]. The methods of approach and detection of the unroofed portion were variable. Zaikokuji et al. performed total endoscopic repair via a right fourth intercostal approach with 3-cm skin incision using a Nelaton catheter passed from the CS to the left atrium [2]. Bozso et al. employed an inferior semicircular periareolar approach though the third intercostal space and, using a 5-mm endoscope search through the CS, visualized the unroofed portion at the CS terminal within the left atrium [3]. Onan et al. performed a totally endoscopic and robotic operation, whereby the precise location of the lesion was detected by direct robotic vision [4].

**Table 1:** Previous reports about minimally invasive URCS repair

Author	Year	Age sex	Diagnosis (type)	Approach	Method of detection	End point
Zaikokuji et al. [2]	2015	54 M	URCS (III)	Total endoscope	Nelaton catheter	
Bozso et al. [3]	2016	49 F	URCS (IV)	Total endoscope and periareolar approach	Endoscopic search	Home
Onan et al. [4]	2016	35 F	URCS (IV) + Secondum ASD	Total endoscope and robot	Vision of the robot	Home
Current case	2020	77 F	URCS (III)	Endoscope	Preoperative 3D-TEE	Home

*URCS* unroofed coronary sinus, *ASD* atrial septal defect, 3*D* three-dimensional, *TEE* transesophageal echocardiography

In the present case, no tension was applied to the surrounding tissue by the direct suture, and the unroofed portion could be directly closed without problem. A pericardial patch might be a good choice when surrounding tissue will be stressed by use of a direct suture.

A minimally invasive approach has a limited visual field and the unroofed coronary sinus may be located in an area that is difficult to directly visualize, as noted in the present case. In the current case, the unroofed portion was detected easily using preoperative 3D TEE; although it could not be recognized intraoperatively by direct vision, it was endoscopically observable. Nowadays, cardiac CT and MRI have become indispensable imaging tools affording excellent anatomical information near the defect and combined lesions [5], while TEE is considered a very useful tool that is capable of detecting precisely the unroofed portion and its anatomical relationship with the orifice of CS [6]. In parallel with the development of the technology, 3D TEE has been increasing in popularity, especially for preoperative and intraoperative analysis of congenital heart disease [7], including URCS [8]. Although cardiac CT and MRI bring us to precise anatomical diagnosis about URCS, these examinations can’t easily build the intracardiac 3D imaging, like a “Surgeon’s view”, which 3D TEE is good at providing. In this point, 3D TEE is superior to cardiac CT and MRI as the preoperative examinations, when surgeons understand the location of URCS intuitively. It is difficult to detect lesions of type III URCS ASD: using a Nelaton catheter passed from the CS to the left atrium allows one to barely recognize the defect [2]. We easily and clearly detected the unroofed portion via the thoracoscope, supported by the preoperative 3D TEE findings. So there was no need using Nelaton catheter. The collaboration of the thoracoscopic approach and 3D TEE for URCS is a promising new method encouraged by the success of our present case. Minimally invasive surgical repair for URCS, which provides the benefit of good postoperative recovery and cosmetic result for patients, seems set to become a feasible approach by exploiting the advantages of 3D cardiac imaging, much the same as has occurred for mitral and tricuspid valve repair.

## Conclusions

The remarkable development of cardiac imaging technologies for URCS, especially 3D TEE, has provided accurate preoperative diagnosis and the ability to detect intuitively the precise location of the unroofed portion. We successfully repaired URCS safely and effectively via a minimally invasive approach directed by cardiac imaging technology, particularly preoperative 3D TEE. This approach promises to become a feasible alternative to conventional techniques.

## Data Availability

The authors declare that all data in this article are available within this published article.

## Abbreviations

3D: Three-dimensional
ASD: Atrial septal defect
CS: Coronary sinus
CT: Computed tomography
MRI: Magnetic resonance imaging
PLSVC: Persistent left superior vena cava
TEE: Transesophageal echocardiography
URCS: Unroofed coronary sinus syndrome
